# Sepsis-Associated Muscle Wasting: A Comprehensive Review from Bench to Bedside

**DOI:** 10.3390/ijms24055040

**Published:** 2023-03-06

**Authors:** Ikumi Yoshihara, Yutaka Kondo, Ken Okamoto, Hiroshi Tanaka

**Affiliations:** 1 Department of Emergency and Disaster Medicine, Graduate School of Medicine, Juntendo University, Tokyo 113-8421, Japan; 2 Department of Emergency and Critical Care Medicine, Juntendo University Urayasu Hospital, Chiba 279-0021, Japan

**Keywords:** muscle wasting, post-intensive care syndrome, sepsis, ICU, critically ill

## Abstract

Sepsis-associated muscle wasting (SAMW) is characterized by decreased muscle mass, reduced muscle fiber size, and decreased muscle strength, resulting in persistent physical disability accompanied by sepsis. Systemic inflammatory cytokines are the main cause of SAMW, which occurs in 40–70% of patients with sepsis. The pathways associated with the ubiquitin–proteasome and autophagy systems are particularly activated in the muscle tissues during sepsis and may lead to muscle wasting. Additionally, expression of muscle atrophy-related genes Atrogin-1 and MuRF-1 are seemingly increased via the ubiquitin–proteasome pathway. In clinical settings, electrical muscular stimulation, physiotherapy, early mobilization, and nutritional support are used for patients with sepsis to prevent or treat SAMW. However, there are no pharmacological treatments for SAMW, and the underlying mechanisms are still unknown. Therefore, research is urgently required in this field.

## 1. Introduction

Sepsis is a leading cause of mortality in intensive care units (ICUs) [1,2]. It is characterized by a very high mortality rate of 20–30%, which further increases to 40–50% following complications, such as respiratory and circulatory failure [3]. Furthermore, sequelae remain even after recovery, and there are many cases in which daily life becomes difficult. However, sepsis has a variety of causes and severity, with many unknown aspects of its pathology.

Sepsis-associated muscle wasting (SAMW) is characterized by decreased muscle mass, reduced muscle fiber size, and muscle strength loss, resulting in persistent physical disability [4]. SAMW is associated with increased morbidity and mortality, and systemic inflammation is reported to be the main cause [5,6]. It occurs in 40% of critically ill, ICU-hospitalized patients and is associated with prolonged ventilator use, extended hospital stay, increased mortality, and long-term functional disorders [7]. In particular, muscle wasting in sepsis occurs early and rapidly during the first 10 days of ICU stay [8]. Furthermore, many critically ill patients who survive are said to have a lower quality of life after hospital discharge due to decreased physical function [9,10]. Thus, although improvement and prevention of SAMW is an important issue, there are no pharmacological therapeutic drugs for SAMW.

In the present review, we outline the pathophysiology, treatment options, and future directions of SAMW.

## 2. Compliance with Ethics Guidelines

This study did not require the approval of an ethical committee because it is a review based on previously published studies. No unpublished data are included.

## 3. Mechanisms and Pathophysiology

### 3.1. Physiological Role of Skeletal Muscle

The skeletal muscle is an important tissue that accounts for approximately 40% of the total body weight; it is the largest tissue in the human body. Furthermore, skeletal muscle is responsible for many functions in the human body, such as movement, maintaining posture, breathing, and protecting internal organs. Skeletal muscle is composed of discrete muscle fiber types defined by myosin heavy chain (MyHC) isoforms and metabolic activity: type I (slow twitch) fibers with slow oxidative ability and type II (fast-twitch) fibers with fast oxidative and glycolytic ability, with each having specific metabolisms and contraction patterns [11].

Type I fibers have a rich capillary supply, a high number of mitochondria and aerobic respiratory enzymes, and a high myoglobin concentration. In contrast, Type II fibers have a low mitochondrial number, high ATP activity, and increased strength and shortening speed on muscle. The proportion of type I and II fibers is variable according to the condition of the human body. Thus, many researchers have investigated the ability of fiber types to transition from slow to fast and vice versa. Of note, skeletal muscle serves as a protein reservoir used in life-threatening situations, such as starvation and severe diseases, including sepsis.

### 3.2. Mechanisms of Muscle Wasting

Muscle wasting occurs systemically as a physiological response to aging and many systematic diseases, including trauma, burns, and sepsis; muscle atrophy occurs in specific muscles with inactivity or denervation [12]. In skeletal muscle, three major pathways are known to be involved in muscle wasting.

The first is the ubiquitin–proteasome system, which plays a key role in muscle mass loss and is involved in the upregulation of ubiquitin-conjugating enzymes (E2) and ubiquitin–protein ligases (E3). Muscle atrophy gene-1 (Atrogin-1; also known as MAFbx) and muscle ring finger-1 (MuRF1) were the first muscle-specific ubiquitin ligases to be discovered [13], and they are now key target genes for muscle wasting.

The second is the calpain system, which belongs to the calcium-dependent cysteine protease family [14]. The calpain system is involved in myofibrillar protein consumption. Furthermore, an in vivo study showed that the administration of calpain inhibitors reduced muscle atrophy by 30% [15]. The calcium-activated calpains are considered modulator proteases because their limited proteolytic activity alters the structure and function of the target substrate.

The third is the autophagy system, a cell catabolic process that ensures the breakdown and restoration of cellular components. Although autophagy has been found to play an important role in maintaining muscle homeostasis and, in practice, may contribute to muscle degeneration, it is a necessary mechanism for cell survival. Nevertheless, increased autophagy activities have been reported to contribute to muscle loss under various conditions, including cancerous cachexia, chemotherapy, disuse, fasting, denervation, and even sepsis [16,17].

The mechanisms underlying muscle wasting, including these three pathways, have not been fully elucidated, warranting further research.

### 3.3. Specific Mechanisms of SAMW

Muscular wasting is a major complication of sepsis and occurs in 40–70% of patients with sepsis [2]. The progression of muscle wasting greatly influences clinical prognosis [18,19]. Inflammatory cytokines such as IL-6, TNF-α, IFN-γ, and IL-1β, whose expression levels increase at the onset of sepsis, cause acute muscle wasting [12,20,21,22,23]. Among inflammatory cytokines, IL-6 has also been reported to directly affect myofibrils [24]. Inflammatory cytokines activate many signaling pathways involved in muscle protein degradation or promote muscle atrophy-related gene expression. Additionally, other factors can influence muscle wasting. For instance, the use of a ventilator accelerates muscle atrophy owing to the inactivity of the strength and mass of the diaphragm, which is a crucial respiratory muscle [25].

Inflammatory cytokines suppress the activation of AMPK, which acts as an energy sensor, and activate mTOR and p70S6K, which are involved in protein synthesis located downstream. However, inflammatory cytokines simultaneously activate the JAK/STAT and PI3K/Akt pathways, which are involved in protein degradation in the ubiquitin–proteasome system, activate the expression of the muscle atrophy-related genes Atrogin-1 and MuRF1, and induce muscle atrophy. They are also known to activate the p38MAPK/NF-kB transduction pathway, which is involved in the inhibition of skeletal muscle differentiation and muscle protein degradation.

**Figure 1:**
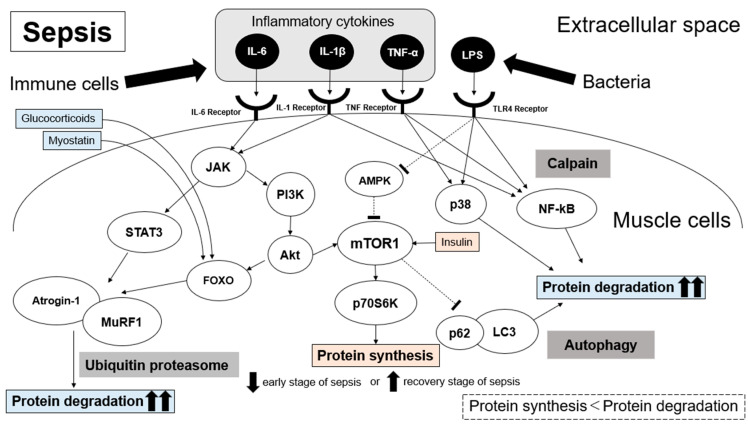
Proposed molecular mechanisms underlying sepsis-related muscle wasting. Immune cells release inflammatory cytokines and activate the ubiquitin–proteasome, calpain, and autophagy signaling pathways. Protein degradation effects overwhelm protein synthesis, and muscle wasting develops. Dotted lines indicate inhibition.

Thus, inflammatory cytokines activate a number of degradative pathways, which result in protein degradation exceeding protein synthesis, leading to muscle wasting in sepsis. The pathways of the ubiquitin–proteasome system and autophagy system are reported to be particularly active during sepsis [26,27,28,29]. In particular, muscle atrophy-related genes Atrogin-1 and MuRF-1 are seemingly increased via the ubiquitin–proteasome pathway [30,31,32]. We have visually summarized the proposed mechanism of SAMW (Figure 1).

### 3.4. Pathophysiology of Muscle Changes in Patients with Sepsis

Histological changes in muscles are mainly evaluated by microscopy with tissue staining, and the muscles may require an objective measure of the muscle fiber mean size, size variation, and types of fibers. Thus, muscle fiber cross-sectional area (CSA) is used as a standard technique for the evaluation of SAMW.

A previous randomized control trial reported a 26% decrease in CSA seven days after the onset of sepsis, and the loss was improved by intensive physiotherapy [33]. In a previous trial, CSA was associated with muscle strength, and it was found that the amount of physiotherapy might lead to better muscle mass maintenance. Furthermore, there are several other studies on the measurement and evaluation of CSA in critically ill patients [34,35].

In contrast, the CSA method can hardly distinguish the types of skeletal muscle fibers, such as type I and type II. An enzyme histochemical staining for NADH-tetrazolium reductase, myosin ATPase, and cytochrome C oxidase is required to classify type I and type II. Only a few studies have focused on muscle fiber types in patients with sepsis [36,37,38]. An observational study revealed an average daily decrease in CSA of 4% for type II skeletal muscle fibers and 3% for type I skeletal muscle fibers in the anterior tibialis muscle of patients with sepsis [37]. Moreover, loss of the filamentous structure of myosin occurred before the degradation of actin or cytoskeletal proteins and was associated with increased expression of lysosomal enzymes and ubiquitin.

In another study with muscle biopsies of the vastus lateralis, CSA was significantly reduced in type IIa and type IIb fibers in critically ill patients, including those with sepsis [38]. The changes in CSA of type II fibers are reduced already early in treatment in the ICU. In addition, significantly lower transcript levels of MyHC isoforms were observed in the muscle.

### 3.5. Effect of Lipopolysaccharides on Skeletal Muscle Cells

Lipopolysaccharides (LPS) bind to genes present on the surface of immune cells and induce inflammatory reactions through the production of inflammatory cytokines via intracellular signal transduction; LPS are also called endotoxins. The receptor for LPS is the toll-like receptor 4 (TLR4). When bound to LPS, TLR4 is transported to CD14 on the plasma membrane, which acts as a co-receptor for TLR4, and activates the expression of MyD88, a cellular protein adapter. MyD88 activates the NF-kB signaling pathway, which promotes protein degradation via the ubiquitin–proteasome system; thus, LPS administration induces an inflammatory response. Myoblasts, particularly the C2C12 line, are often used as an in vitro model in research focusing on muscle wasting. Previous research reported that adding LPS to C2C12 myoblasts increases the mRNA levels of the inflammatory cytokines TNF and IL-6 in a dose-dependent manner [39]. IL-6 has also been shown to decrease myotube diameter in C2C12 cells, and the expression of Atrogin-1 and MuRF1 has been reported to increase with IL-6 expression [24]. Moreover, the addition of LPS to C2C12 cells promoted the production of IL-1β, suggesting that IL-1β may be directly involved in muscle fiber atrophy [40].

### 3.6. Effect of Cecum Ligation and Puncture on Experimental Animals

Sepsis models are often used in animal experiments by ligating the cecum and inducing intraperitoneal infection with its contents to induce peritonitis in mice (cecum ligation and puncture; CLP). Many studies on sepsis and muscle wasting have been reported in experiments conducted using CLP model mice (Table 1) [24,41,42,43,44,45,46,47,48,49,50,51]. Among previous studies (10/12, 83.3%) evaluated muscle wasting within a week after CLP. Morphological changes of muscle wasting were seen from 2 to 24 days after the CLP procedure. Additionally, morphological changes were mainly assessed by histological evaluation, and some studies (4/12, 33.3%) included the results of weight in the muscles. Various muscles were found to be wasting after CLP, including the tibialis anterior, gastrocnemius, soleus, extensor digitorum longus, diaphragm, and heart muscle. Many studies (7/12, 58.3%) reported that the tibialis anterior muscle was mainly wasted after CLP, indicating that the tibialis anterior muscle is the most easily influenced muscle during sepsis and underlying sepsis-related muscle wasting.

### 3.7. Differences between Disuse Muscle Atrophy and SAMW

Disuse muscle atrophy can be detected as early as 1 week after inactivity, whereas SAMW can be detected as early as 2 days after onset; therefore, disuse muscle atrophy and muscle atrophy resulting from sepsis may have different mechanisms [52,53,54]. Additionally, type II fibers have been found to be affected more than type I fibers in SAMW, whereas disuse of muscles more easily affects type I fibers [41,42]. Providing evidence that type II fibers are easily affected in SAMW, it has been reported that mTOR, which controls the muscle protein synthesis system, is suppressed in the skeletal muscle during the onset of sepsis. However, the signal transduction may occur only in type II fibers [50,55,56]. The FoxO genes, activated by sepsis, are located upstream of MuRF1 and Atrogin-1 and regulate downstream muscle atrophy-related genes (Table 1). It has also been reported that FoxO-related muscle atrophy is mainly prominent in type II skeletal muscle fibers [41,57].

Furthermore, recoveries from disuse muscle atrophy and SAMW differ remarkably. A previous study has reported that mTOR and its downstream muscle protein synthesis-related genes are more activated than in controls at 12 to 24 h following re-loading after disuse muscle atrophy [58]. Thus, recovery of muscle protein from disuse muscle atrophy takes place in a relatively short period of time, whereas SAMW recovery takes a long time and is less likely to return to before-sepsis conditions. This is because SAMW is not merely a reduction in muscle protein but is deeply debilitating due to sustained activation of protein degradation pathways, such as the ubiquitin–proteasome system [59].

### 3.8. Muscle Wasting, Particularly Diaphragm Wasting in Sepsis

SAMW occurs in both skeletal muscles and the diaphragm, presenting specific electrophysiologic and morphologic findings. However, the underlying mechanisms differ, and here we mention some specific characteristics of muscle wasting in the diaphragm.

Mechanical ventilation is an important treatment option for a life-threatening event, and many sepsis patients require mechanical ventilation for respiratory support. However, ventilator-related diaphragm wasting is caused by excessive power of artificial breathing and may lead to worse clinical outcomes. Although most patients can be weaned from the ventilator, 30% of critically ill patients cannot avoid extended use of mechanical ventilation [60]. A prior study has reported that approximately 50% of patients have decreased diaphragm muscle thickness after intubation [61]. Both decreased and increased diaphragm thickness in the early course of mechanical ventilation predicted prolonged ventilation. Decreasing thickness of diaphragm was related to very low inspiratory effort, and increasing thickness was related to excessive effort [62]. Furthermore, a prolonged period of mechanical ventilation has been reported to be associated with an increased risk of death and worse long-term outcomes. Fewer than half of patients could not survive beyond a year, although a high proportion of patients could be discharged from the hospital [63].

## 4. Diagnostics

In clinical settings, SAMW has been diagnosed using anatomical evaluations and functional tests. Anatomical evaluation is performed using muscle biopsy followed by a histological exam, computed tomography (CT), magnetic resonance imaging (MRI), and ultrasonography.

Muscle biopsy followed by histological evaluation is considered a highly accurate method to diagnose myopathic changes of SAMW. However, the biopsy method can be accompanied by some complications such as bleeding, pain, and nerve injury; therefore, alternative diagnostic tools have been considered instead.

A CT scan is widely accepted as the gold standard method for skeletal muscle mass quantification. An observational study using a CT scan reported on the measurement and evaluation of the rectus femoris muscle in patients with sepsis [64]. The measurement was confirmed at the vertebral level of L4 on the CT scan; the area of the psoas major muscle was traced in 2 to 4 cuts, depending on the thickness of the CT slice. The technique was also used in the rectus femoris muscle to assess muscle volume [64].

MRI is also used for diagnoses of SAMW and, similar to CT, has a highly accurate diagnostic value for muscle mass [65]. However, an MRI scan takes a long time, and most metallic devices are contraindicated based on major concerns regarding the powerful magnetic field generated by MRI. Thus, patients who undergo MRI scan need to be hemodynamically stable.

Ultrasonography is easy to use, with almost no complications, and therefore can become a useful diagnostic option for SAMW. Recently, many studies focused on ultrasonography for evaluating mass volume in sepsis instead of MRI and CT imaging. The authors of a study using ultrasonography measured muscle thickness of the rectus femoris muscle over time after the admission of patients with sepsis [66]. The ultrasonography method could reveal that rapid muscle wasting started early during hospitalization, and muscle thickness continued to decrease from day 3 to day 10 [66]. Other studies using ultrasound to measure rectus femoris muscle thickness reported a decrease in muscle thickness of approximately 10% during the ICU stay [67] and a 1.45% decrease in the CSA of the rectus femoris muscle per day [68]. In addition to muscle thickness, alterations in muscle echotextures in the early stages of sepsis also have been reported [69]. Patients with sepsis are generally not so easy to move to CT or MRI rooms because of the severity of disease; therefore, ultrasonography is recommended for the diagnosis of SAMW.

Functional tests are also useful for evaluating SAMW because muscle volume does not always correlate with muscle strength. Thus, handgrip strength, the medical research council (MRC) scores, and the functional independence measure (FIM) are widely used for assessing SAMW [70]. Regarding the MRC score, muscle strength is graded as follows in 12 skeletal muscle groups: 0, “no visible or palpable contraction;” 1, “visible or palpable contraction without limb movement;” 2, “movement of the limb, but not against gravity;” 3, “movement against gravity;” 4, “movement against moderate resistance;” 5, “movement against complete resistance (normal)” [71]. The total score ranges between 0 and 60, and the sum score < 48 points indicates “muscle weakness.” The FIM consists of 18 items assessing six areas of function, and each item is graded from 1 (total assistance needed) to 7 (total independence) points. The final sum score ranges from 18 (lowest) to 126 (highest).

MicroRNAs (miRNAs) may become a potential biomarker of SAMW although further evidence is required. Innate and adaptive immunity associated miRNA regulates the TNF and the TLR/NF-kB signaling pathway in sepsis [72]. A study reported that myo-miRNA (c-miR-486) and inflammation-related miRNA (c-miR-146a) in plasma may serve as a predictive biomarker of muscle wasting [73].

## 5. Risk Factors

There are some risk factors in SAMW. Sepsis patients often have decreased insulin resistance and have shown hyperglycemia. Moreover, increased levels of insulin resistance and hyperglycemia easily cause SAMW. Thus, sepsis patients often require insulin administration, and insulin can activate mTOR1 which promotes muscle synthesis. Glucocorticoid use is also one of the risk factors of SAMW. Muscle wasting due to glucocorticoids is triggered by the activation of ubiquitin–proteasome system and the catabolic effect may differ with sepsis severity. Avoiding use of glucocorticoid can prevent SAMW. Myostatin may be associated with increasing SAMW, although checking serum myostatin levels is not popular in current clinical settings. Myostatin is both produced and released by monocytes and promote muscle wasting through the ubiquitin–proteasome system (Figure 1). Avoiding those risk factors can be useful for preventing SAMW.

## 6. Treatments

There are no established pharmacological treatments for improving SAMW. Thus, we present several physiological interventions that are clinically used for preventing or improving SAMW.

### 6.1. Electrical Muscular Stimulation

Electrical muscular stimulation (EMS) is commonly used as a part of strength training in the fields of orthopedics and sports medicine; it uses electrical stimulation to force muscle contraction. Passive electrical stimulation of inactive muscles and active electrical stimulation of voluntary muscles can be used for task-specific rehabilitation [74]. It has also been suggested that the early introduction of EMS may contribute to reducing muscle atrophy in ICUs [75,76,77]. Nevertheless, previous studies on the effects of EMS in patients with sepsis showed conflicting results [78,79]. Low-frequency (35 Hz) electrical stimulation was ineffective in maintaining muscle mass, whereas high-frequency (100 Hz) electrical stimulation increased muscle strength [78,79]; therefore, frequency of EMS may have a role in preventing SAMW.

Animal experiments have suggested that EMS improves muscle mass and reduces markers of muscle atrophy and apoptosis [80]. EMS is expected to effectively improve disused muscle atrophy in patients hospitalized in the ICU, where muscle atrophy is attributed to long-term bedridden conditions and progresses with the transition from type I to type II muscle fibers [41,42].

However, muscle atrophy resulting from sepsis causes significant atrophy of fast-twitch fibers, requiring specific and effective fast-twitch fiber stimulation. Recruitment of more motor units is required for the recovery of fast-twitch fibers.

### 6.2. Physiotherapy and Mobilization

Physiotherapy and early mobilization during ICU care are known to be effective in reducing functional decline due to many diseases [81]. It has been reported that physiotherapy has an improvement effect regarding the following three points.

The first is bedrest conditioning. Many studies have shown that long-term bedrest causes many physiological changes and ailments [82]. Additionally, muscle atrophy progresses at a very high rate since sepsis itself promotes protein degradation and inhibits protein synthesis. The second is the suppression of the activation of mechanisms leading to sarcopenia. It has been suggested that sepsis and sarcopenia have the common risk factor of aging [83], and although sarcopenia usually progresses with aging, it is also known to be accelerated and exacerbated by diseases. The third is an increase in lung and tissue aerobic capacity. Several studies have reported that physical therapy and early mobilization interventions ameliorate the above-mentioned issues related to ICU care. In previous studies, physiotherapy and early mobilization were shown to reduce the number of days on a ventilator [77,81], shorten the duration of hospital stay [84], and improve functional capacity at hospital discharge [85,86,87]. Furthermore, physiotherapy within 90 days of hospitalization is associated with the risk of death 10 years later [88].

### 6.3. Nutritional Support

Patients hospitalized in the ICU experience accelerated systemic protein degradation. Clinical research has suggested that nutritional therapy plays a major role in disease outcomes and improvement [89,90]. Some advocate that high protein intake (1.5–2.5 g/kg per day) for critically ill patients contributes to improving some clinical outcomes compared with conventional protein intake (~0.8 g/kg per day) [91,92].

Several studies have focused on muscle fiber type shifts and nutrition. First, type II fibers are said to undergo significant muscle protein degradation during starvation owing to malnutrition [93]. At the onset of sepsis, a starvation response by autophagy occurs in the body, indicating that muscular atrophy resulting from sepsis causes significant type II fiber atrophy. Type II fibers use sugars such as glycogen as an energy source, and consumed glycogen takes approximately 24–48 h to be resynthesized. High carbohydrate intake may increase the recovery rate from type II fibers loss via rapid glycogen synthesis [94].

Leucine, an essential amino acid, has also been reported to provide nutritional support for muscle synthesis. Leucine is the main component of muscle fibers, and its function is to increase insulin secretion, helping muscle cells uptake glucose as an energy source. By promoting insulin secretion, leucine increases endurance and explosive power during exercise, promoting muscle growth, repair, and strength after exercise [95].

Since the underlying mechanisms of the disease differ between patients, these nutritional therapies cannot be applied uniformly to all patients. We should provide nutrition for patients with sepsis, considering the patient’s condition and nutritional balance.

### 6.4. Pharmacological Intervention and Future Directions

Currently, EMS, physiotherapy, early mobilization, and nutrition support are conducted for preventing and treating SAMW in clinical practice; however, no drug therapy has been found. A new treatment method for SAMW using pharmacological therapy has been eagerly anticipated.

Hibernations have some organ protective effects, although the cellular and molecular basis of mammalian hibernation remains poorly understood. The proportions of monounsaturated fatty acids in the muscles of hibernating animals are higher during hibernation, suggesting an increased ability to utilize fat tissues for energy [96]. To prevent muscle atrophy, hibernating animals increase the reabsorption rate of urea from their urine, which decreases the necessity to use amino acids by degrading protein from skeletal muscles [97]. Some mammals also retain the hibernation gene, referred to as the hibernation-specific protein; it has been reported that this protein is produced in the liver and acts on the brain during hibernation [98]. Hibernation-specific proteins work to overcome the winter months and starvation by switching to a low metabolic state [99,100]. Hibernation is characterized by a dormant period lasting from several days to several weeks, depending on the species, in which the basal metabolic rate drops to 2–4% of normal conditions, and the body temperature is maintained at a few degrees above ambient temperature [101,102]. Such hypothermia and hypometabolism lead to irreversible cell membrane damage and loss of cellular ionic homeostasis in critical organs, such as the brain and heart in humans and most mammals, which do not retain hibernation genes and cannot withstand prolonged hypothermia and hypoxia. In contrast, drug-induced hibernation, “artificial hibernation,” may maintain homeostasis of the human body by adjusting doses of the drug and keeping moderate hypothermia.

The hibernation effect could become a treatment option for SAMW through the above-suggested mechanisms. A drug-induced hibernation effect, namely “artificial hibernation,” may prevent and treat SAMW.

**Figure 2:**
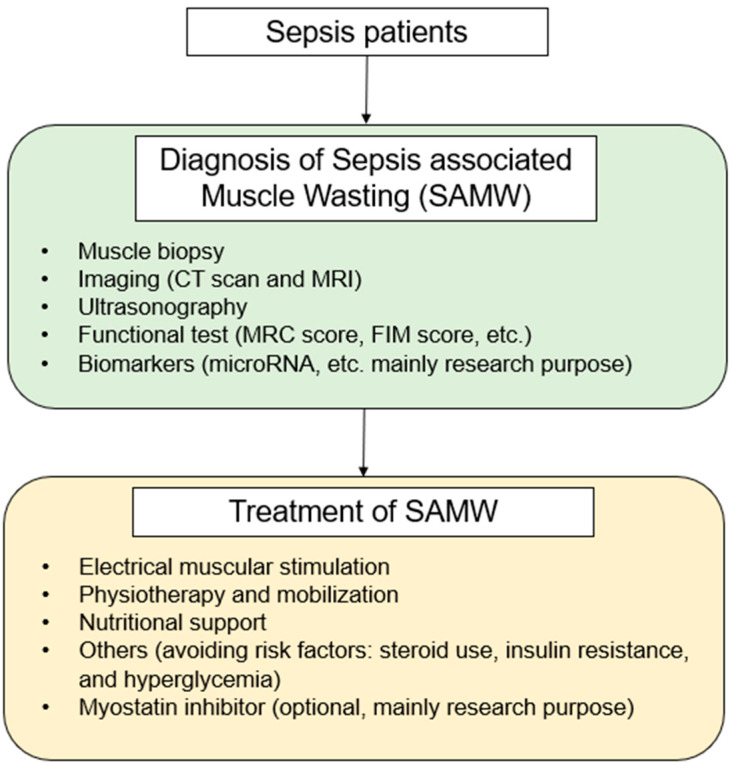
Flow chart of sepsis-related muscle wasting for diagnosis and treatment.

We have shown a summary flow chart of SAMW (Figure 2).

## 7. Conclusions

Muscle wasting resulting from sepsis develops in 40–70% of patients with sepsis; it is a clinically important complication that greatly affects the exacerbation, recovery, and prognosis of sepsis. Muscle proteins throughout the body deplete rapidly during the initial stage of sepsis. EMS, physiotherapy, early mobilization, and nutritional support are clinically used for the purpose of preventing or treating SAMW. Future research for treatment focused on SAMW is warranted.

## Figures and Tables

**Table 1:** Characteristics of CLP studies for muscle wasting.

No.	First Author, Year	Country	Mouse/Rat	Sepsis Model	Evaluation Days after Onset of Sepsis (Day)	Treatments/Gene Knock Out	Doses of Treatment	Timing of Treatment	Antibiotics	Muscle Wasting in the Control Group	Muscle Wasting in the Treatment Group	Weight of Muscles Changes	Grip Strength	Histology	Atrogin-1 Expression in Muscles in the Treatment Group	MuRF1 Expression in Muscles in the Treatment Group	Reference No.
1	Reed SA, 2012	US	C57BL/6 mouse	CLP	7	-	-	-	(−)	TA, GAS, SOL muscle wasting (+)	-	-	-	-	-	-	[41]
2	Morel J, 2017	France	C57BL/6 mouse	CLP	7	-	-	-	(−)	GAS, Diaphragm muscle wasting (+)	-	-	-	-	-	-	[42]
3	Balboa E, 2018	US	C57BL/6 mouse	CLP	7	-	-	-	(−)	GAS, TA muscle wasting (+)	-	-	-	-	-	-	[43]
4	Yu X, 2018	China	C57BL/6 mouse	CLP	1~7	Heme Oxygenase-1	50 mg/kg	1 day before CLP	(−)	SOL muscle wasting (+), ≥3 days after CLP	SOL muscle wasting (−), ≥3 days after CLP	improved	Not described	Improved	Decreased	Decreased	[44]
5	Moarbes V, 2019	Canada	C57BL/6 mouse	CLP	1~4	-	-	-	(−)	TA, Diaphragm muscle wasting (+)	-	Not described	Not described	-	-	-	[45]
6	Wang J, 2020	China	SD rats	CLP	24	Testosterone propionate	10 mg/kg	8 days after CLP	(−)	EDL muscle wasting (+)	EDL muscle wasting (−)	Not described	Not described	Improved	Not described	Not described	[46]
7	Kobayashi M, 2021	Japan	C57BL/6 mouse	CLP	14	Myostatin-deficient	-	-	(−)	TA, GAS, SOL muscle wasting (+)	TA, GAS, SOL muscle wasting (−)	Not described	Not described	Improved	Decreased	Decreased	[47]
8	Busch K, 2021	Germany	NLRP3 knock out mouse	CLP	4	NLRP3 knock out	-	-	(−)	Heart muscle wasting (+)	Heart muscle wasting (−)	improved	Not described	Improved	Not described	Not described	[48]
9	Yang B, 2022	China	C57BL/6 mouse	CLP	2	IL-6 knock out	-	-	(−)	EDL muscle wasting (+)	EDL muscle wasting (−)	Not described	Improved	Improved	Decreased	Decreased	[49]
10	Yin D, 2022	China	SD rats	CLP	3	Neuregulin-1β	10 µg/kg	12 h after CLP	(−)	TA muscle wasting (+)	TA muscle wasting (−)	Not described	Not described	Improved	Not described	Not described	[50]
11	Jiang Y, 2022	China	C57BL/6 mouse	CLP	5	Limb-immobilization	-	with CLP	(−)	TA muscle wasting (+)	TA muscle wasting (+)	deteriorate	Deteriorate	Deteriorate	Not described	Not described	[51]
12	Zanders L, 2022	Germany	IL6 knock out mouse	CLP	1~4	IL-6 knock out	-	-	(−)	TA muscle wasting (+), morphological changes were seen in 4 days after CLP	TA muscle wasting (−), morphological changes were seen in 4 days after CLP	improved	Not described	Improved	Decreased	Decreased	[24]

CLP: Cecal ligation and puncture, US: United States of America, TA: Tibialis anterior, GAS: Gastrocnemius, SOL: Soleus, EDL: Extensor digitorum longus, SD: Sprague-Dawley, NLRP3: NLR family pyrin domain containing 3.

## Data Availability

Not applicable.

## Abbreviations

ICU: intensive care unit; SAMW: sepsis-associated muscle wasting; MyHC: myosin heavy chain; MuRF1: muscle ring finger-1; CSA: cross-sectional area; LPS: lipopolysaccharides; TLR: toll-like receptor; CLP: cecum ligation and puncture; CT: computed tomography; MRI: magnetic resonance imaging; MRC: medical research council; FIM: functional independence measure; EMS: electrical muscular stimulation.
